# Human traffic and habitat complexity are strong predictors for the distribution of a declining amphibian

**DOI:** 10.1371/journal.pone.0213426

**Published:** 2019-03-07

**Authors:** Rachel B. Anderson

**Affiliations:** Entomology Department, University of California, Davis, Davis, California, United States of America; Universidade Federal de Goias, BRAZIL

## Abstract

Invasive species and habitat modification threaten California's native pond-breeding amphibians, including the federally threatened California Red-legged Frog (*Rana draytonii*). The relative contributions of invasive species, including the American Bullfrog (*Lithobates catesbeianus*), and of habitat changes to these declines are disputed. I conducted a field study over several years in central California to examine the presence/absence of these two species at 79 breeding ponds to determine the predictive role for occupancy of factors including vegetation, pond characteristics, and measures of human activity. I used a boosted regression tree approach to determine the relative value of each predictor variable. Increased measures of human activity, especially proximity to trails and roads, were the best predictors for the absence of California Red-legged Frogs and California Newts. Historical factors and habitat conditions were associated with the extent and spread of the American Bullfrog. The extent and complexity of aquatic macrophytes and pond surface area were good predictors for the presence of these and other amphibian species. Surprisingly, invasive species played a relatively small role in predicting pond occupancy by the native species. These findings can inform conservation and restoration efforts for California Red-legged Frogs, which apparently persist best in small vegetated ponds in areas of low human disturbance.

## Introduction

Invasive species represent a serious threat to the world’s biodiversity and to human activities [1,2]. They often have strong dispersal and reproduction abilities, such that established species introductions are frequently irreversible [3,4]. Their negative impacts can include decimating naïve native species through competition or predation. However, some native species can mitigate these impacts by changing their behavior or habitat use in the presence of invasive predators. Habitat management and creation to enable this type of separation may provide the most feasible and cost-effective solution to promote native amphibian populations and counter invasive species’ threats in many systems [1,5]. Studies of the habitat associations of native and invasive taxa can inform these management efforts.

Habitat complexity that creates spatial or abiotic variation in the environment can allow a greater number of species to coexist by altering which species has a competitive advantage over space and/or time [6,7]. An environmental gradient can ensure that one species is unable to competitively dominate another similar species, provided they have different sensitivities to abiotic variation [8]. Variation in resource distribution in space can also influence the outcome of competitive interactions [9]. Habitat structure can limit the effects of predators as well: increased structure and complexity can reduce the feeding efficiency of predators [10,11].

Pond-breeding anurans provide a good system to test the relative impact of invasive species and habitat factors because of ease of observation and the fact that these amphibians often occur in relatively isolated populations, with and without invasive species present. Globally-invasive American Bullfrogs (*Lithobates catesbeianus*) are well documented as predators and competitors of other amphibian species at all life stages [8,12–14]. Both larvae and adults may induce microhabitat avoidance and increased hiding in their amphibian prey, which reduces feeding opportunities for these intraguild prey [15]. Similarities in natural history between American Bullfrogs and other large ranids (e.g., long larval periods and highly aquatic adults) suggest that interactions should be greatest among these species.

American Bullfrogs were introduced for food and bait into the western United States and have spread widely; they have strong impacts on native amphibian species, particularly other large ranids [16–18]. American Bullfrogs are particularly abundant in lowland or degraded habitats, which coincide with areas from which the California Red-legged Frog (*Rana draytonii*) has disappeared [16,17,19]. The California Red-legged Frog is a federally listed threatened species, and is declining due to factors that include introduced American Bullfrogs and fish [20]. Some evidence suggests that California Red-legged Frogs survive poorly in the presence of American Bullfrogs [13,17], but apparent coexistence has been observed in a few areas [21].

Habitat complexity at both the breeding pond and landscape scales can limit the effects of intra- and interspecific competition among large ranids. Field studies indicate that small, human-modified permanent ponds with less shallow water and little emergent vegetation support fewer native ranids [9,22], whereas highly complex habitats with large areas of shallow water and a large extent of emergent vegetation may ameliorate negative interactions, thus supporting more and larger populations of endemics [9,19,23]. This type of habitat contributes to higher water temperatures and algal productivity in the shallows, benefitting eggs and tadpoles, and the spatial heterogeneity allows use of different microhabitats by different post metamorphic age classes [15].

Intact upland habitat, without human development or roads, may benefit native amphibian populations. Land use adjacent to ponds is known to affect amphibian distribution and abundance; the presence of many species is correlated with increased natural cover and negatively correlated with road density and percent impervious surface [24,25]. These more natural landscapes can benefit amphibian populations in two ways. Intact terrestrial upland habitat may allow for greater refuge habitat for juveniles and increasing foraging habitat for adults [21,26,27]. Vegetated corridors may also allow for successful dispersal of individuals and support metapopulation dynamics, whereas landscape alteration can contribute to habitat fragmentation and isolation of breeding ponds [5,28,29]. Effects of different cover types, and adequate riparian buffer requirements, can be species-specific [30]. Changes to land use can benefit introduced species, including the American Bullfrog, which is known to disperse easily through human-altered landscapes, where it uses edge habitats, constructed roads, and permanent wetlands as corridors to travel up to 7km in 4 d [19,22,31,32].

Decline of California's native amphibians has been reported for decades, and is attributed to land use changes as well as to species introductions [16,19]. California Red-legged Frogs were also over-harvested in market hunts prior to the early 1900s [18]. Human-caused changes may work together to present an ongoing threat to these and other native species [32]. In particular, introduction of the American Bullfrog has been cited as a cause of decline for many California species, though its relative role compared to human land use has been disputed [20]. Using several years of field surveys, I recorded biotic and abiotic habitat variables for amphibian breeding ponds, as well as amphibian community compositions. I used these data to test to what extent California Red-legged Frog presence is predicted by the presence of the invasive American Bullfrog as well as by pond and landscape-level habitat factors.

## Materials and methods

**Fig 1:**
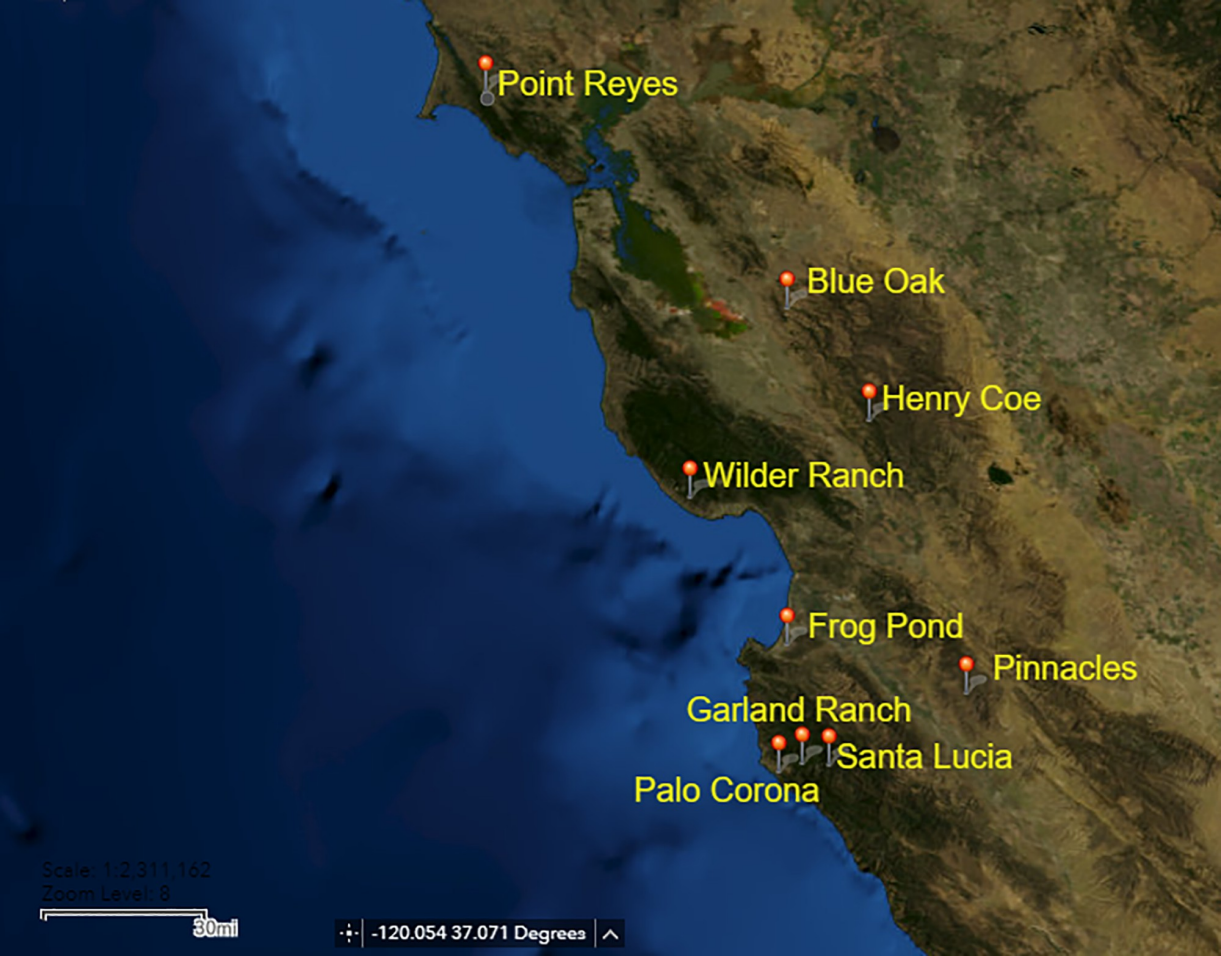
Survey locations. Field sites for a study of native California Red-legged Frogs (*Rana draytonii*) and invasive American Bullfrogs (*Lithobates catesbeianus*), examining the role of habitat factors in coexistence or exclusion of these two species. Created using USGS National Map Viewer.

I identified 79 study ponds on public lands in central California of four types: 38 uninvaded ponds with California Red-legged Frogs only, 17 ponds where California Red-legged Frogs and American Bullfrogs coexist, 14 ponds with only American Bullfrogs, and 9 ponds with neither species (S1 Table). I included only ponds for which amphibian survey data existed for at least the previous 10 yr, to verify presence or absence of amphibian species. Sample sizes for each condition were limited by availability of past data and accessibility of study areas. Ponds were geographically interspersed and separated by >2km. During the site selection process, land managers provided initial confirmation of species composition at these ponds via personal communication and previous biologist reports. Ponds were distributed across ten study locations that consisted of parks and other reserves, extending from Point Reyes National Seashore in the northeast to Pinnacles National Park in the southwest, including multiple locations in the Monterey-Carmel region (Fig 1). This study area encompasses most of the current range of the California Red-legged Frog; populations in southern California and in the Sierra foothills have almost entirely disappeared [33]. I conducted surveys during 2013, 2014, 2015, and 2016, repeating surveys across years when possible (S1 Table). Previous amphibian surveys exist for these ponds and areas for the past 10–15 years; these reinforced the presence data collected in this survey. Historical records describing species composition at any specific pond site before American Bullfrog invasion do not exist, and thus determining whether California Red-legged Frogs have been extirpated from individual sites is not possible. Surveys were conducted under USFWS Permit TE63330, California Department of Fish and Wildlife permit SC-11950, and appropriate authorization from land owners or administrators.

I conducted amphibian surveys using several standard methods [34]. These included auditory surveys, visual encounter surveys, and dipnet sampling. The use of multiple survey techniques results in a more complete characterization of the amphibians present in a pond than single-method surveys [35]. When possible, surveys began in February, during the breeding season of California Red-legged Frogs, and were repeated monthly through September, to observe the breeding and metamorphosis of California Red-legged Frogs and of American Bullfrogs. I visited each site for at least one day and night survey, each at least one hour in duration, usually completed on the same date. Day surveys were conducted between 10:00 and 16:00, and night surveys from one hour after sunset until 02:00, to limit fluctuations in temperature. For both day and night surveys, I conducted auditory surveys to identify species of calling male frogs, as well as visual encounter surveys to record all amphibian individuals observed along the shores of ponds. I walked along the perimeter while scanning the bank and on the entire surface of the water with and without binoculars. I sampled larval amphibians using a standard dipnet procedure [36,37]. I swept a D-shaped dipnet with 1.8-m handle length perpendicular to the bank, along the substrate, beginning about 1.5m out. I spaced these net samples roughly evenly around the perimeter, with at least 10m between sweeps. This technique provides a fast, thorough sample with minimal effort and time [38]. All amphibians were returned to the pond after sampling.

At each site, I noted physical descriptors including latitude, longitude, and elevation. I measured pond characteristics including area and average and maximum water depth. I noted the primary substrate (e.g. silt, sand) and pond type (natural, or type of construction; most artificial ponds were constructed for cattle) and when ponds dried, if they were not permanent. I classified ponds as ephemeral or permanent based on whether they dried completely each year. I noted water color, and rated turbidity from 1 (completely clear) to 5 (completely turbid). I estimated extent of center, edge, submerged, and floating vegetation, which included aquatic macrophytes and woody species, as well as extent of algal growth and canopy cover. I classified vegetation into guilds according to overall size and branching structure: none, graminoids, low forbs, high forbs, cattail/tule, and branching shrubs. I ranked the relative abundance of each guild in each area. I also recorded presence of other aquatic vertebrates including common species that may indicate habitat suitability for amphibians such as the Pacific Chorus Frog (*Pseudacris sierra)*, Western Toad (*Anaxyrus boreas*), and Western Pond Turtle (*Actinemys marmorata*), the endangered or species of special concern California Tiger Salamander (*Ambystoma californiense*) and California Newt (*Taricha torosa*), and predators including garter snakes (*Thamnophis sirtalis* or *T*. *elegans*), wading birds, Western Mosquitofish (*Gambusia affinis*), and non-native centrarchid fishes (*Micropterus sp*, *Lepomis sp*).

Metrics of human influence included distances to trails and roads. These measurements were obtained from satellite imagery, measured as a straight-line distance from the center of each pond to the nearest trail, dirt road, and paved road. I also included a habitat condition scale assessing modification of the habitat. This 1–5 scale follows Fellers and Freel (1995), with 1 a relatively inaccessible pristine habitat with no sign of human disturbance, 3 representing a body of water with nearby recreation (e.g. trails, picnic tables) and a 5 an entirely artificial pond in an urban landscape [37]. Habitat was assessed for a 150m buffer around each pond—the maximum distance typically moved by non-migrating California Red-legged Frogs [39–41].

I used a boosted regression tree (BRT) approach to assess the importance of each of these habitat and other variables to the presence/absence of California Red-legged Frogs and of American Bullfrogs. Boosted regression trees use a combination of two approaches: regression trees, which predict the value of a response based on a series of binary splits constructed from multiple input variables, and boosting, which builds and combines many simple models to improve predictive ability [42]. Each consecutive tree is fitted to the residuals of the preceding tree, thus building on the variation not already explained by the model. The final BRT model is similar to an additive regression model in which each individual term is a simple tree [42]. BRTs incorporate advantages of tree-based approaches, including its ability to handle different types of predictor variables and to accommodate missing data [42], while increasing predictive performance by fitting multiple trees, each to a random subset of data [43].

I included all landscape, biotic, and human variables described above as predictors (Table 1). I used the packages 'gbm' (Ridgeway 2017) and 'dismo' (Hijmans et al 2017) in R to calculate the contribution of each predictor variable to model fit, averaged across all trees. To fit a BRT, I modified values of two parameters—tree complexity (number of nodes) and learning rate (shrinkage parameter)—to find minimum predictive error. Tree complexity (tc) determines the level of interaction that can be fitted: a tc value of 1 allows for one node (decision rule), a value of 2 indicates 2 nodes and allows 2-way interactions, etc [44]. Learning rate determines the contribution of each tree to the growing model, such that a smaller learning rate increases the number of trees required [45]. A model with the highest possible tree complexity and smallest learning rate is considered preferable [42].

**Table 1:** Selected predictors used in the boosted regression tree (BRT) analysis of presence/absence of California Red-legged Frogs (*Rana draytonii*) and American Bullfrogs (*Lithobates catesbeianus*) in a field survey in Central California.

Predictor (units)	Measurement/How defined	Mean ± SD (where applicable)
*Area (m*^*2*^*)*	Surface area of pond at survey	4669.21 ± 13900.05 m^2^
*AvgDepth (m)*	Estimate of average pond water depth	1.59 ± 1.17 m
*Bullfrog*	Presence (1) or absence (0) of American Bullfrog, *Lithobates catesbeianus*	
*CalNewt*	Presence (1) or absence (0) of California Newt, *Taricha torosa*	
*Canopy Cover (%)*	Estimate of % water surface with tree cover	6.01 ± 11.75%
*CenterVeg (%)*	Estimate % surface area of pond occupied by floating or emergent macrophytes	31.96 ± 32.04%
*CenterVegSp1*	Guild of most abundant vegetation type on water surface	
*CenterVegSp2*	Guild of second most abundant vegetation type on water surface	
*Condition*	Metric of human modification of pond, ranked 1 (pristine, inaccessible) to 5 (urban, heavily modified)	
*DirtRoadDistance (m)*	Direct distance to nearest dirt road	67.94 ± 137.55 m
*Elevation (m)*	Elevation of surveyed pond	301.96 ± 233.33 m
*Latitude (DD)*	Latitude of pond	
*Longitude (DD)*	Longitude of pond	
*MaxDepth (m)*	Measure of pond maximum water depth	2.40 ± 1.44 m
*PavedRoadDistance (m)*	Direct distance to nearest paved road	626.66 ± 841.68
*TrailDistance (m)*	Direct distance to nearest hiking trail	34.77 ± 51.37 m
*Turbidity*	Water clarity rated 1 (perfectly clear) to 5 (no visibility)	
*Vegetated Edge (%)*	Estimated % of pond perimeter with vegetation	76.58 ± 30.29%
*VegetatedEdgeSp1*	Guild of most abundant vegetation type on pond perimeter	
*VegetatedEdgeSp2*	Guild of second most abundant vegetation type on pond perimeter	
*WaterColor* *WhenDry*	Metric of algal extent: 1: clear, 2: present, 3: extensive green, 4: dark/bright green Classified by water permanency or ephemerality	

I varied tree complexity from 1 to 5 and learning rate from 0.05 to 0.01. I accepted as final models those that generated at least 1000 trees without increasing deviance (overfitting to training data) [42]. The gbm output gives the relative contribution of each predictor, scaled so that the sum of these adds to 100. I also used the output to find and visualize interactive effects of main predictors on amphibian presence. I used the function ‘rcorr’ in the package ‘Hmisc’, which computes a matrix of Pearson’s r for all possible pairs of predictors, to examine the correlation structure for the predictor variables. Although the BRT framework accounts for correlations (because outputs reflect the relative contribution of each predictor with all others held at their mean), it is informative to explore covariance among factors because of their potential biological importance. I used the output of the correlation matrix to examine potential relationships between scaled and continuous variables hypothesized to vary together. These included latitude and longitude, metrics of human impact, and pond conditions, including turbidity, water color, water permanency/ephemerality, and area. I considered correlations significant at P < 0.05. For all analyses, I used R version 3.4.1 (R Development Core Team, 2017).

## Results

**Fig 2:**
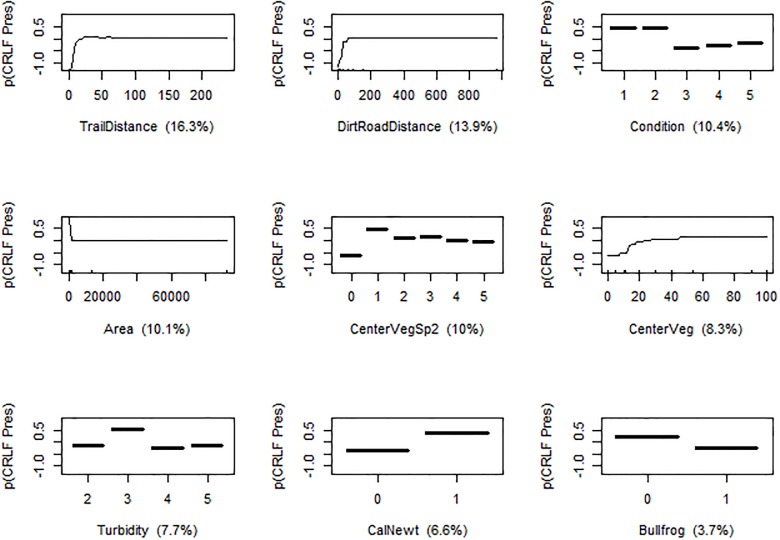
Partial dependence plots for the nine most influential variables in the boosted regression tree (BRT) model predicting probability of California Red-legged Frog (CRLF; *Rana draytonii*) presence in ponds. Predictors were measured during a field study of California Red-legged Frogs in Central California. Relative importance is depicted for each predictor, holding all other predictors at their mean. See Table 1 for units and explanations of each predictor.

The best model of California Red-legged Frog presence at a pond included metrics of human influence, vegetation conditions, and species interactions, which combined to contribute 87.0% to the final model (Fig 2). The most powerful predictor was distance to trail (relative contribution to final model 16.3%), with increased distance increasing probability of California Red-legged Frog presence. Increasing distance to nearest dirt road showed the same effect (contribution 13.9%). Habitat condition, a metric of human modification, predicted California Red-legged Frog presence (10.4%), with frogs more likely found in ponds of condition 1 or 2 (i.e. least disturbed habitat). These three metrics together contributed to 40.6% of the final model.

Local pond conditions and other species comprised the remaining influential factors. Smaller surface area correlated with California Red-legged Frog presence (10.1% contribution to final BRT). Species diversity of emergent and floating vegetation was important: California Red-legged Frogs were more likely to be found in ponds with more than one plant guild on the surface as compared to a single type (10.0%). Increasing extent of aquatic vegetation increased probability of California Red-legged Frog presence (8.3%). Moderate turbidity was associated with California Red-legged Frog presence in the model (7.7%). Only two of the recorded vertebrate community species played predictive roles: California Red-legged Frogs were more likely to be found in ponds that were also occupied by the California Newt (6.6%), and were less likely to be found in ponds with American Bullfrogs (3.7%).

**Fig 3:**
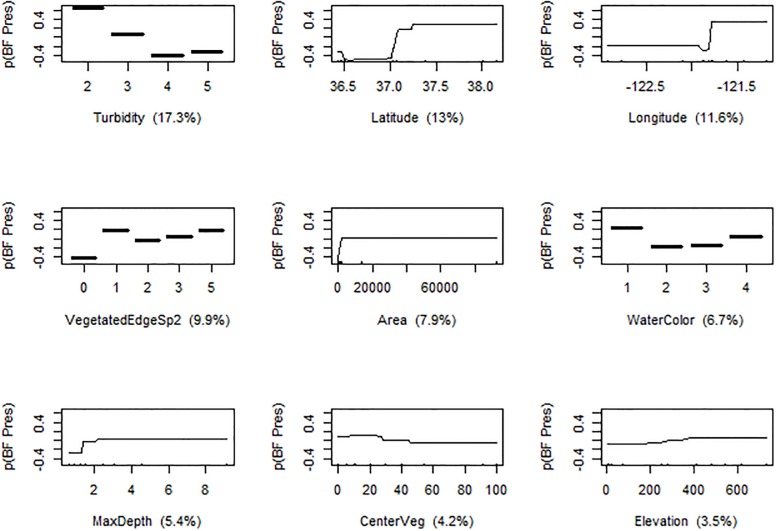
Partial dependence plots for the nine most influential variables in the boosted regression tree (BRT) model predicting probability of American Bullfrog (BF; *Lithobates catesbeianus*) presence in ponds. Predictors were measured during a field study of California Red-legged Frogs (*Rana draytonii*) in Central California. Relative importance is depicted for each predictor, holding all other predictors at their mean. See Table 1 for units and explanations of each predictor.

**Fig 4:**
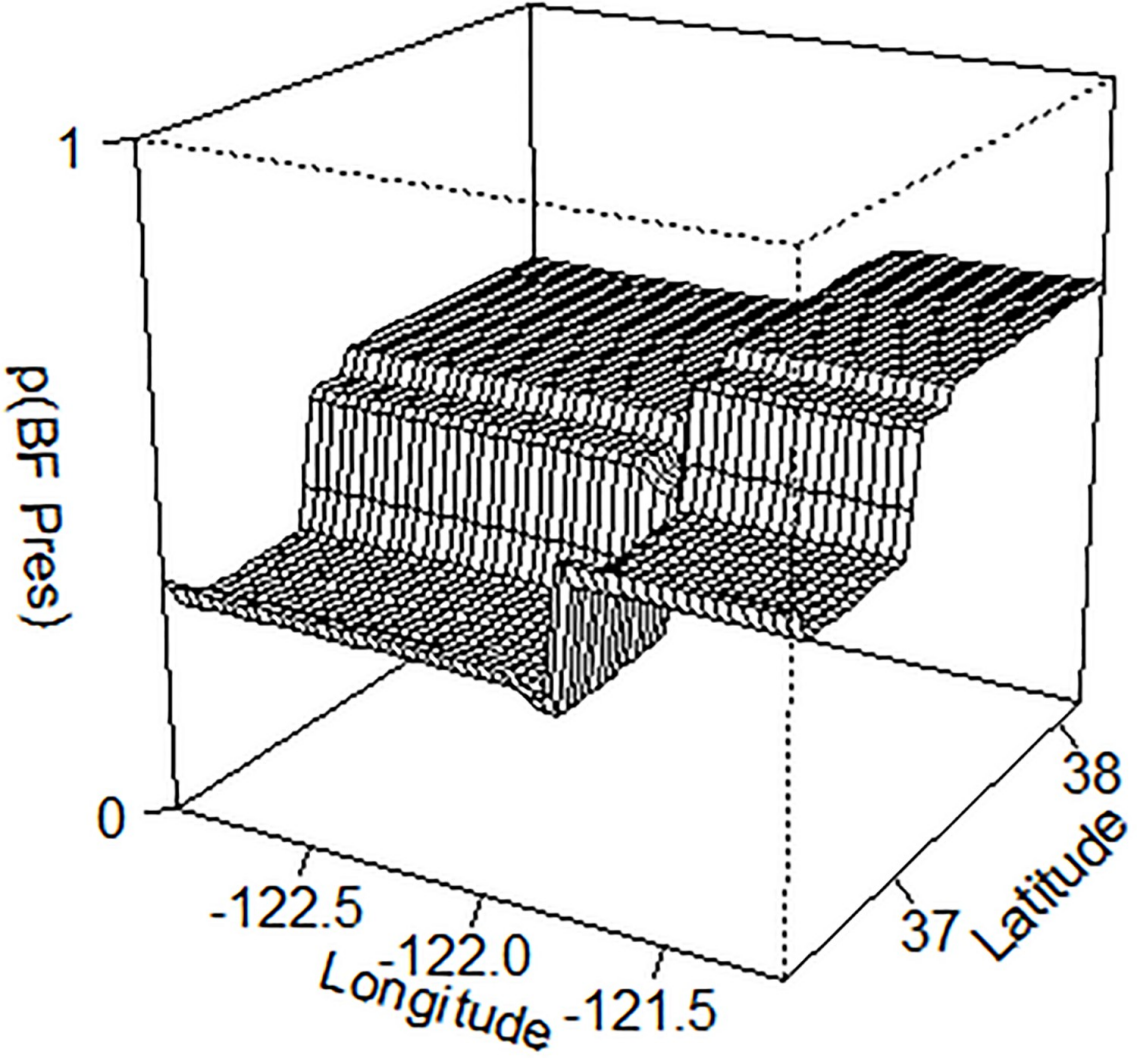
Three-dimensional partial dependence plot for the interaction of longitude and latitude in predicting American Bullfrog (BF; *Lithobates catesbeianus*) presence at a pond, using data collected during a field study in Central California. Longitude (West-East) on x-axis, latitude (South-North) on y-axis, probability of American Bullfrog presence on z-axis. American Bullfrog presence is most likely in the northeast and least likely in the southwest of the study region, indicating historical and modern release sites.

The most powerful predictors of American Bullfrog presence at a pond included local pond conditions as well as geographical descriptors, together related to 79.5% of variance in the final BRT (Fig 3). The most powerful predictor was turbidity, with American Bullfrogs more likely to be found in less turbid waters (turbidity contributed 17.3% to the final BRT). American Bullfrogs were also more likely to be found at lower latitudes (13%) and at more western sites (longitude contributed 11.6%). The BRT analysis reported an interactive effect of longitude and latitude on American Bullfrog distribution (Fig 4). Species diversity of the perimeter vegetation affected occupancy: American Bullfrogs were more likely to be found in ponds with more than one plant guild on the perimeter as compared to a single type or bare shores (9.9%). Pond size was also a strong predictor in the BRT, with American Bullfrogs more likely found in ponds with greater surface area (7.9%). Water color, a metric of algal extent, also predicted American Bullfrog occupancy: clearer waters corresponded to presence (6.7%). American Bullfrogs were more likely to occur in deeper ponds according to the BRT model (5.4%). A moderate (20–40% coverage) extent of aquatic vegetation predicted American Bullfrog presence (4.2%). Increased elevation (from 0 to 600m) corresponded with American Bullfrog presence (3.5%).

Some metrics of human activity and of local pond conditions correlated significantly. The measure of “condition” (ranked 1–5, with 5 representing most degraded habitats) was negatively correlated with distance to trail (r = -0.299, p = 0.007), distance to dirt road (r = -0.336, p = 0.002), and distance to paved road (r = -0.337, p = 0.002). However, these distances were not always significantly correlated (e.g. distance to trail and distance to paved road, r = -0.072, p = 0.528). Increased turbidity was correlated with ephemeral habitats (r = 0.315, p = 0.005). A clearer water color had a significant relationship with greater maximum water depth (r = 0.243, p = 0.030).

## Discussion

### Predictors for California Red-legged Frog presence

For California Red-legged Frogs, metrics of human influence proved to be the most important variables in determining presence at a given pond. Overland distance to hiking trails, and to dirt and paved roads were some of the most powerful predictors, indicating that human foot and vehicle traffic may be a significant detriment to these frogs. Though they are highly aquatic, they are known to make sometimes lengthy overland movements [40,41]. Additionally, proximity to road could correlate with other human use impacts (e.g. pollution, changes in hydrology and runoff). Previous work showed that California Red-legged Frog presence at a breeding pond was negatively affected by both road proximity and number of roads within a 500m buffer [22]. That study examined only paved roads, whereas the present study included trails and dirt roads, indicating that California Red-legged Frogs may face multiple threats from human traffic. These unpaved roads affect populations at a smaller spatial scale: the average distance to trail and dirt road for ponds with California Red-legged Frogs was 44.80 and 24.41m, respectively, whereas trails and dirt roads averaged 11.78 and 16.92m from ponds without California Red-legged Frogs (also see Fig 2). The present study did not examine road density, which may play an important role in determining impact of human activity. Changes in terrestrial habitat can affect amphibian occupancy at a smaller scale than factors that affect dispersal and metapopulation persistence (e.g. road density) do [30]. California Red-legged Frogs were also more likely to be found at ponds that were less disturbed, inaccessible via trail (backcountry), or slightly affected by current human use, e.g. a created wetland (cattle pond) on now-protected land. This pattern could represent modern impacts, including increased hiding behavior and habitat degradation, as well as historic extirpations due to hunts for frogs as food [18]. A study of human impacts on Iberian frogs (*Rana iberica*) found that frog abundance decreased with proximity to recreational areas and recommended the establishment of 2.5m buffers to limit loss of spatial and temporal availability of resources [46].

Local pond conditions including structure and complexity of aquatic macrophytes as well as pond size also positively predicted California Red-legged Frog presence. Vegetation extent is known to be important for amphibian breeding and occupancy [5,21]. In particular, more vegetation on the surface of a pond, including emergent and floating vegetation, was important for California Red-legged Frog presence. For California Red-legged Frogs, emergent vegetation serves as oviposition sites, while larval habitat includes floating vegetation and cattails [47]. In addition to greater surface coverage overall, diversity of center vegetation was important: having more than one guild of vegetation within a pond (e.g. both floating pondweed, *Potamogeton natans* and tule, *Schoenoplectus acutus*) predicted California Red-legged Frog presence. This additional structure may have provided more microhabitat for use by different life stages of the California Red-legged Frog, and may have allowed for resource partitioning and protection for recently metamorphosed juveniles from conspecific adults, which are known to consume smaller frogs [15,48]. Pond surface area was negatively correlated with California Red-legged Frog presence; these were more common in smaller ponds (median area of occupied ponds 232.3, range 2.3–23225.8 m^2^). Small ponds may have less human influence and fewer introduced species, and stay warmer for faster growth of eggs and larvae [27]. California Red-legged Frogs also commonly occupied more turbid ponds, and higher turbidity was in turn correlated with earlier seasonal drying. Ephemeral ponds may be more productive seasonally, and can exclude fish and American Bullfrogs, which require permanent water.

Some species interactions played a minor role in explaining California Red-legged Frog presence: occurrences were positively correlated to presence of the California Newt and only weakly negatively related to presence of the American Bullfrog. California Newts may have overlapping habitat requirements with the California Red-legged Frog: both are aquatic spring breeders, and adults use aquatic environments but also make overland movements [49]. Previous studies have indicated the importance of buffer zones from human disturbance for pond-breeding salamanders [50,51]. California Newts may additionally be more likely to be found with California Red-legged Frogs because they prey on amphibian eggs and larvae [49]. Although American Bullfrog presence was negatively related to California Red-legged Frog presence, this invader played a smaller predictive role than expected. American Bullfrogs are known to prey upon and compete with California Red-legged Frogs, so studies of presence/absence may not to reflect the full impact on populations at a pond [15] or could fail to describe changes in metapopulation dynamics. Further study of density of native amphibians at ponds with and without invasive American Bullfrogs should help to resolve the impact of American Bullfrog presence on California Red-legged Frog populations and should reveal habitat correlates of increased survival of natives. Fish presence was not a predictor for amphibian occupancy in this study, though fish are known to be important in amphibian distribution [52]. Low variability in fish presence at surveyed ponds may have limited predictive ability in this study—only 8% of ponds contained game fish. The presence of crayfish similarly lacked sufficient variance to be meaningful. Twelve percent of ponds contained *Gambusia*, which may have limited effects on large amphibians [13].

### Predictors for American Bullfrog presence

American Bullfrog presence in Central California correlated with several local pond habitat factors, including pond size. American Bullfrogs were more likely to be found in bigger, deeper, water bodies, likely because they hold water long enough for their year-long larval period [53]. Greater pond depth corresponded to clearer water color (less algal growth), which was a positive predictor for American Bullfrog presence. Metrics of human activity were not factors in predicting American Bullfrog presence: these frogs are known to be tolerant of urbanization and changes to the landscape, including other introduced species [54].

Pond location played an important role in determining American Bullfrog presence, including latitude and longitude. American Bullfrogs were most likely to be found in the northeast region of the study area, and least likely to be found in the southwest areas. This likely reflects historical patterns of introduction as well as natural barriers to modern spread of the invasion. American Bullfrogs were repeatedly introduced to California cities from 1900–1930 to meet a demand for frog legs for human consumption [18], and are still imported into city centers (e.g. San Francisco and environs, in the northeastern region of this study) for this market [55]. However, Storer in 1925 reported American Bullfrogs from many central California counties, including Monterey (in the southwestern portion of the study area), so failure of American Bullfrogs to spread through ponds in these areas likely reflects presence of barriers to colonization [56]. These barriers could include scarcity of permanent ponds, which limits successful reproduction. Other studies have shown that American Bullfrogs are more successful at lower elevations, perhaps due to ease of dispersal through human-modified landscapes or their low cold tolerance [56,57], but topography did not appear to be a limiting factor for American Bullfrogs in this study.

American Bullfrog presence was correlated with few microhabitat or vegetation covariates but they were more likely to be found in ponds with vegetation diversity along the water's edge. Ponds with more than one guild of vegetation on the pond perimeter tended to support American Bullfrogs, perhaps by protecting recently metamorphosed juveniles [58]. Additionally, American Bullfrogs were more likely found in ponds with decreased surface vegetation. Large amounts of emergent vegetation may hinder movements of this highly aquatic frog, or lack of vegetation may be correlated with larger pond area and increased depth (preventing growth of species that must root in the substrate).

Unexpectedly, American Bullfrogs appeared to play only a minor negative predictive role in California Red-legged Frog presence at a potential breeding pond. One explanation may be that presence of the two anuran species correlated with largely non-overlapping habitat predictors, indicating that the species are using bodies of water with different characteristics in this region of California. Large bodies of water in human-dominated areas are likely to be unusable by California Red-legged Frogs because of unsuitable habitat characteristics regardless of American Bullfrog presence, so removal of American Bullfrog in developed areas may have little benefit to California Red-legged Frog populations. One exception could be a large urban lake linked to a nearby small protected pond, where American Bullfrogs could use the ephemeral habitat to forage and thus create a sink habitat for the native.

#### Management implications

These findings have implications for conservation of the endangered California Red-legged Frog. The present study affirms the results of previous studies that have shown the importance of upland habitat for adults [40,41]. Adults can occupy terrestrial habitats extensively during the dry season and traverse among ponds, but may have difficulty with anthropogenic barriers [42]. They may be unable to avoid nearby roads, devegetated areas, or other obstacles that prove fatal to them, especially in areas highly modified by human use. It is also possible that frogs are subject to capture or harassment when near trafficked areas. This study indicates that even trails or dirt roads can have negative impacts on occupancy. Small, remote ponds that are likely to be occupied by the California Red-legged Frog and the California Newt should be protected against increased human activity and development, including the building of hiking trails within a biologically relevant distance. Previous work has shown that the density of paved roads within a 500m buffer negatively impact amphibian populations [22,42]; land managers should consider a buffer of at least 50–100m to limit the impact of trail and dirt roads.

These findings can also be used to guide active management via the creation of new pond habitat for California Red-legged Frogs. Created wetlands can benefit native species if they are a suitable distance from human traffic, while remaining within colonization distance from existing populations (e.g. <2km for the California Red-legged Frog) [39–41]. Managers should avoid developing trails within a 100m buffer of breeding ponds, and paved roads within a 500m buffer [22]. Additionally, these ponds should remain relatively small and with the potential for seasonal drying (in this study, ponds with California Red-legged Frogs averaged 1500m^2^ in surface area) and with sufficient perimeter and surface vegetation of multiple types. If these created or existing ponds are managed for cattle, fencing or other exclusion should be used to maintain vegetation diversity, particularly of emergent and floating species.

Because native species are often limited to those habitats that are currently appropriate for them, conservation efforts should focus on preserving protected areas, creating and restoring protected ponds similar to occupied habitat, and maintaining suitable pond habitat for existing populations. Evidence from this study suggests that the distribution of American Bullfrogs in this region may be limited by natural barriers to spread beyond historical and modern introduction sites, though further study is needed to determine an estimate of invasion rate. Thus, removal of American Bullfrogs from certain sites (e.g. urban centers surrounded by roads) where they currently occur may be a misdirected conservation attempt. Removal from other habitats more suitable for California Red-legged Frogs may allow re-establishment of natives in those areas, especially where vegetation and hydrology is also planned and managed to benefit native species [59,60]. A recent study found that American Bullfrogs use constructed water catchments as habitats and for dispersal into habitats previously inaccessible to them [61]. Thus, artificial habitats could be removed to limit spread of invaders across the landscape—though connectivity should be maintained for native species. Before costly removal efforts are undertaken for American Bullfrogs or other invaders, habitat suitability or potential for restoration for native species should be assessed. Managers and individuals should avoid introducing American Bullfrogs and other invasive species to smaller, remote, more natural sites where native species thrive, and monitor existing wetland sites.

## Supporting information

S1 Table Study sites and years. Survey years for parks and ponds included in a field study of presence/absence of California Red-legged Frogs (CRLF; *Rana draytonii*) and American Bullfrogs (BF; *Lithobates catesbeianus*) in Central California. Includes presence of large anuran species, either *R*. *draytonii* (CRLF), *L*. *catesbeianus* (BF), both present (both), or neither (none).
(PDF)
