# Supplementary material for: Human traffic and habitat complexity are strong predictors for the distribution of a declining amphibian
Source: PLoS One. 2019 Mar 7;14(3):e0213426. doi: 10.1371/journal.pone.0213426 (PMC6405065; doi:10.1371/journal.pone.0213426)
Supplement: S1 Table — Survey years for parks and ponds included in a field study of presence/absence of California Red-legged Frogs (CRLF; Rana draytonii) and American Bullfrogs (BF; Lithobates catesbeianus) in Central California. Includes presence of large anuran species, either R. draytonii (CRLF), L. catesbeianus (BF), both present (both), or neither (none). (PDF) [file pone.0213426.s001.pdf]

S1 Table. Study sites and years. Survey years for parks and ponds included in a field study of presence/absence of California Red-legged Frogs (CRLF; *Rana draytonii*) and American Bullfrogs (BF; *Lithobates catesbeianus*) in Central California. Includes presence of large anuran species, either *R. draytonii* (CRLF), *L. catesbeianus* (BF), both present (both), or neither (none).

**Park**	**Pond**	**CRLF/BF Presence**	**Years Surveyed**
Blue Oak Ranch Reserve, Santa Clara Co.			
	Big Lake	BF	2013, 2014
	Cabin	Both	2013, 2014
	West	BF	2013, 2014
	Windmill	CRLF	2013, 2014
Frog Pond Wetland Preserve, Monterey Co.			
	Del Monte Lake	None	2013, 2014
	Frog Pond	None	2013, 2014, 2015, 2016
	Laguna Grande	None	2013, 2014
	Lake El Estero	None	2013, 2014
	Monterey Pines	None	2013, 2014
	Roberts Lake	None	2013, 2014
Garland Ranch Regional Park, Monterey Co.			

	Fern	Both	2013, 2014, 2015, 2016
	Mesa	BF	2013, 2014, 2015, 2016
	Veeder	CRLF	2013, 2014, 2015, 2016
Henry Coe State Park, Santa Clara Co.			
	C-24	None	2016
	C-25	Both	2016
	C-26	BF	2016
	C-27	None	2016
	C-30	CRLF	2016
	C-35	CRLF	2016
	C-41	CRLF	2016
	C-43	CRLF	2016
	C-44	CRLF	2016
	C-59	CRLF	2016
	C-60	Both	2016
	C-62	Both	2016
	C-67	Both	2016
	C-68	BF	2016
	C-76	CRLF	2016
Palo Corona Regional Park, Monterey Co.			
	Animas	CRLF	2013, 2014, 2015, 2016

	Boundary	none	2013, 2014, 2015, 2016
	Dead Pig	CRLF	2013, 2014, 2015, 2016
	Entrance	both	2013, 2014, 2015, 2016
	Roadrunner	none	2013, 2014, 2015, 2016
	Salamander	CRLF	2013, 2014, 2015, 2016
	River	CRLF	2015, 2016
Pinnacles National Park, San Benito Co.			
	BGR	CRLF	2013, 2014
Point Reyes National Seashore, Marin Co.			
	Estero Pond	CRLF	2013
	Estero Trailhead	CRLF	2013
	Five Brooks	both	2013
	Giacomini 1	CRLF	2013
	Giacomini 2	CRLF	2013
	Haigmeir	BF	2013
	Marshall Beach Trailhead	CRLF	2013
	Mud Lake	both	2013
	Olema Hill	both	2013
	Olema Marsh	both	2013

	Tomales Bay	BF	
	Trailhead		2013
Santa Lucia Preserve, Monterey Co.			
	GR Pond	CRLF	2013, 2014, 2015
	LG-15	BF	2013, 2014, 2015
	LG-16	BF	2013, 2014, 2015
	LG-17	CRLF	2013, 2014, 2015
	LG-18	BF	2013, 2014, 2015
	LG-19	both	2013, 2014, 2015
	LG-20	both	2013, 2014, 2015
	LG-21	CRLF	2013, 2014, 2015
	LG-22	BF	2013, 2014, 2015
	LG-23	both	2013, 2014, 2015
	LG-24	BF	2013, 2014, 2015
	PO-01	CRLF	2013, 2014, 2015
	PO-02	CRLF	2013, 2014, 2015
	PO-03	CRLF	2013, 2014, 2015
	PO-04	CRLF	2013, 2014, 2015
	PO-05	CRLF	2013, 2014, 2015
	PO-06	none	2013, 2014, 2015
	RO-10	CRLF	2013, 2014, 2015
	RO-11	CRLF	2013, 2014, 2015

	RO-12	CRLF	2013, 2014, 2015
	RO-13	CRLF	2013, 2014, 2015
	RO-14	both	2013, 2014, 2015
	SC-25	BF	2013, 2014, 2015
	SC-26	both	2013, 2014, 2015
	SC-27	CRLF	2013, 2014, 2015
	SJ-08	CRLF	2013, 2014, 2015
	SJ-09	none	2013, 2014, 2015
Wilder Ranch State Park, Santa Cruz Co.			
	Ag Pond	CRLF	2013, 2014
	Dimeo	CRLF	2013, 2014
	RR1	CRLF	2013, 2014
	RR2	CRLF	2013, 2014
	RR3	CRLF	2013, 2014
